# Venous Thromboembolism Associated with Uterine Fibroids: A Review of Reported Cases

**DOI:** 10.3390/jcm15020444

**Published:** 2026-01-06

**Authors:** Radmila Sparić, Marta Stojković, Momir Šarac, Giovanni Pecorella, Vladimir Živković, Safak Hatirnaz, Andrea Tinelli

**Affiliations:** 1 Faculty of Medicine, University of Belgrade, Dr Subotića 8, 11000 Belgrade, Serbia; radmila@rcub.bg.ac.rs (R.S.); mstojkovic037@gmail.com (M.S.); vladimir.zivkovic@med.bg.ac.rs (V.Ž.); 2 Clinic for Gynecology and Obstetrics, University Clinical Centre of Serbia, Dr Koste Todorovića 26, 11000 Belgrade, Serbia; 3 Faculty of Medicine of the Military Medical Academy, University of Defence, Crnotravska 17, 11000 Belgrade, Serbia; dr.momirsarac@gmail.com; 4 Clinic for Vascular and Endovascular Surgery, Military Medical Academy, Crnotravska 17, 11000 Belgrade, Serbia; 5 Department of Obstetrics and Gynecology, and CERICSAL (CEntro di RIcerca Clinico SALentino), “Veris delli Ponti Hospital”, Via Giuseppina Delli Ponti, 73020 Scorrano, LE, Italy; giovannipecorella2690@gmail.com; 6 Institute of Forensic Medicine, Faculty of Medicine, University of Belgrade, Deligradska 31a, 11000 Belgrade, Serbia; 7 School of Health Sciences, Kent University, Istanbul 34433, Türkiye; safakhatirnaz@gmail.com; 8 Department of Obstetrics and Gynecology, Mediliv Medical Center, Samsun 55100, Türkiye

**Keywords:** uterine fibroids, leiomyoma, pelvic mass, venous thromboembolism, deep vein thrombosis, pulmonary embolism, complication, case reports review

## Abstract

**Background/Objectives**: The most prevalent benign tumors in women are uterine fibroids. Most patients—more than half—do not exhibit any symptoms, but the most common clinical signs include irregular uterine bleeding, pelvic pain, gastrointestinal problems, increased frequency of urination, and, in some cases, infertility. Venous thromboembolism is a very rare consequence, especially when significant uterine fibroids are present. This syndrome usually develops because of pelvic vascular systems being compressed, which causes venous stasis. Pharmacological treatment, minimally invasive procedures, and surgical techniques are examples of therapy alternatives. The purpose of this study is to present, compare, and potentially elucidate the underlying mechanisms of VTE development in fibroids. **Methods**: we have synthesized findings from 24 documented instances of venous thromboembolism (VTE) linked to uterine fibroids. **Results**: the principal mechanism underlying thromboembolic events was identified as the mechanical compression of pelvic venous structures due to mass effect. Additionally, we recognized other pertinent risk factors, including oral contraceptive use, May-Thurner syndrome, myomatous erythrocytosis, and intravenous leiomyomatosis. None of the reviewed case reports provided evidence of confirmed inherited thrombophilia in the patients under investigation. The femoral and popliteal veins, primarily in the left leg, were most frequently impacted by thrombosis and the ensuing blockage. Imaging techniques confirmed that individuals suffered pulmonary embolisms in half of the cases. When the right treatment was given as soon as possible, most of VTEs had favorable outcome. In almost half of the cases examined, the patient had a hysterectomy. Since all symptoms were alleviated and the chance of additional thromboembolic consequences was reduced, this treatment strategy turned out to be the most successful. **Conclusions**: Clinicians should maintain a low threshold for venous imaging in women with large pelvic masses and unilateral limb symptoms. Despite being uncommon, VTE associated with UFs can cause serious morbidity. Mechanical venous compression is the main mechanism, which is often exacerbated by additional prothrombotic variables. Clinicians should maintain a low threshold for venous imaging in women with significant pelvic masses and unilateral limb symptoms, look for concurrent thrombophilia, and investigate early surgical consultation to address compressive etiologies when VTE is still unexplained. It would be simpler to ascertain the actual incidence and pinpoint risk variables that can be altered with standardized reporting of fibroid-associated VTE and prospective registries.

## 1. Introduction

Uterine fibroid (UF), also known as leiomyoma, is a frequent ailment among women in reproductive age. According to histology, smooth muscle cells from the myometrium—the uterine muscle layer—proliferate monoclonally to cause these benign tumors. Although certain genetic alterations have been found that may contribute to its etiopathogenesis, the exact cause of this proliferation is still unknown [1,2]. Literature identifies early menarche and parity as significant risk factors [3,4]. Additionally, lifestyle factors such as smoking, obesity, BMI, an inadequate diet rich in red meat but deficient in fruits and vegetables, and a lack of physical activity are hypothesized to contribute to the onset of these benign tumors [3,5].

About half of UF sufferers are thought to be asymptomatic. The size, quantity, and position of the fibroids, as well as the existence of coexisting degenerative disorders, all influence the onset and intensity of symptoms. Menorrhagia, dysmenorrhea, and intermenstrual bleeding are examples of abnormal uterine bleeding (AUB), which is the most common symptom. Along with bulk symptoms brought on by the tumor’s growth and pressure on other organs, such as bloating, frequent urination, and bowel problems, pelvic discomfort is also frequently experienced, regardless of menstruation [6,7]. Subfertility is also linked to UFs. A number of variables are thought to be involved, such as altered smooth muscle contractility in the myometrium, uterine cavity deformation, and mechanical compression of the fallopian tubes [8]. Fibroids, as the most prevalent benign tumors in women can also cause miscarriage, fetal growth restriction, low birth weight, fetal malposition and malrotation, premature delivery, placental abruption, and antepartum hemorrhage, among other problems and unfavorable obstetrical outcomes [7,9,10,11]. The highest incidence is observed in women in their early 40s, although it is postulated that symptomatic presentation occurs at this age for tumors that may have developed earlier in life [3]. It is estimated that by the age of 50, leiomyomas will develop in 70% of Caucasian women and up to 80% of African American women [12]. UFs pose a significant challenge and adversely affect the quality of life in symptomatic patients, thereby imposing a substantial burden on the healthcare system. In the United States, the direct and indirect costs associated with UFs amount to approximately 34.4 billion USD annually, while in developed European countries, the annual costs of hospital treatment range between 86 and 348 million USD [13,14]. Women who have severe symptoms of this illness often have lower health-related quality of life (HRQL). The main causes of this decrease are bleeding-related symptoms, but psychological problems including anxiety, despair, fear, concern, and poor self-image also have a big influence. Research has shown that younger patients had much lower HRQL because of physiological differences (such as hormone levels, fibroid size and location), fewer childbearing-related concerns, and prior uterine fibroid experience [15].

The literature describes venous thromboembolism (VTE) as an extremely uncommon but possibly fatal consequence, especially in situations with big UFs. The most common observation is that fibroids can compress the blood vessels in the pelvis and nearby extremities due to their large size and volume, which can result in venous stasis. Polycythemia, relative thrombocytosis brought on by menorrhagia, and an elevated generation of procoagulant substances linked to UFs are additional contributory factors [16,17]. Additionally, VTE can occur as a complication of pelvic surgery, specifically myomectomy [18]. This study aims to present, compare, and possibly clarify the underlying causes of the development of VTE in individuals with UFs.

## 2. Materials and Methods

**Figure 1:**
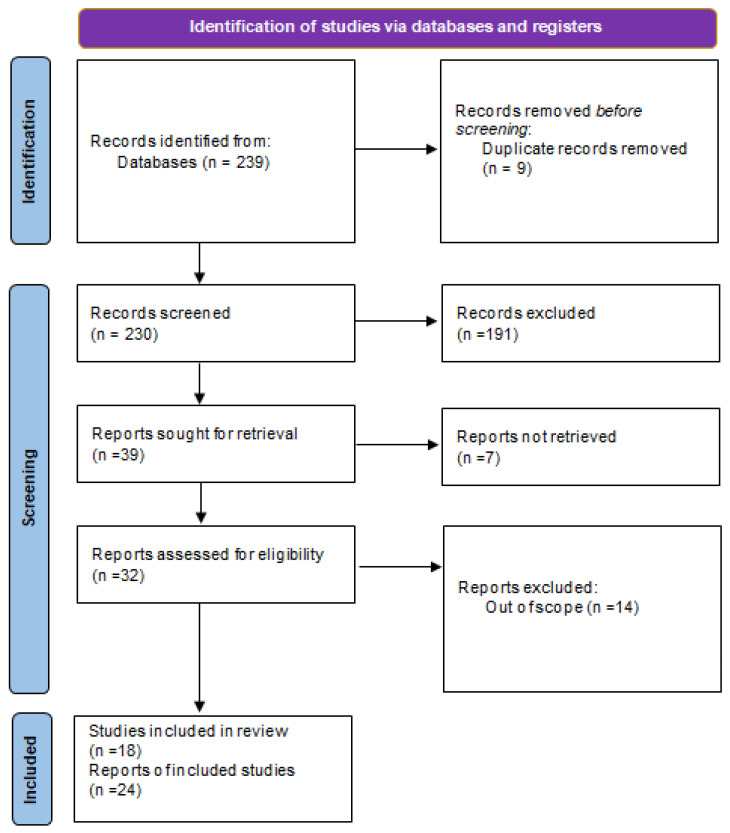
Summary of the literature analysis using the method illustrated in the preparation of the paper.

The investigators undertook a systematic examination of the existing literature concerning women with UFs with past history of thrombosis. A thorough exploration of the MEDLINE, Scopus, and PubMed databases was performed covering the period from 2019 to 2025, employing a strategic amalgamation of search terms, including “uterine fibroids,” “leiomyoma,” “venous thromboembolism,” “pulmonary embolism,” “complication,” and “case report.” Only peer-reviewed scholarly articles detailing clinical instances of thromboembolic incidents in females diagnosed with UFs were incorporated into the review. We searched only papers in English language. A total of 239 records were identified through searches of multiple electronic databases, of various types (case reports, case series, original articles, etc.). After removal of nine duplicate records, 230 studies remained for title and abstract screening. During this phase, 191 records were excluded as they were clearly irrelevant based on title and abstract, including review articles, editorials, and studies that did not report clinical cases. Subsequently, 39 reports were sought for full-text retrieval; however, seven articles could not be retrieved because they were available only as abstracts or conference proceedings. Full-text assessment for eligibility was performed on the remaining 32 reports. Of these, 14 studies were excluded because they did not specifically address the association between uterine fibroids and thromboembolic complications, focused on other pathologies such as fibrosarcoma or ovarian leiomyoma, or were published in languages other than English. Ultimately, 18 studies met the inclusion criteria and were included in the narrative review. Furthermore, supplementary articles were ascertained through the citations found within pertinent studies. A meticulous database investigation was executed by two independent researchers, with any discrepancies being deliberated and resolved through consensus or mediation involving a third researcher. Ultimately, the various discussion topics derived from the literature intended for inclusion in the narrative review were allocated among the contributing authors, followed by collective discourse and examination to facilitate the completion of the manuscript. Through this methodological approach, it was posited that the potential for study selection bias was significantly mitigated. The results of the research have been divided into different paragraphs, with which we have illustrated what has been reported in the scientific literature. In total, 18 studies were included in the review, comprising 24 reported cases of venous thromboembolism in women with UF (Figure 1).

## 3. Results

A total of 24 previously published cases of uterine fibroid-associated venous thromboembolism (VTE) were meticulously examined, involving female subjects aged between 30 and 71 years, with a predominant occurrence in their fourth and fifth decades of life (Table 1). The primary thrombotic presentations encompassed deep vein thrombosis (DVT) and pulmonary embolism (PE), which frequently manifested concurrently. The majority of DVT cases were observed in the left lower extremity, specifically affecting the iliofemoral and popliteal veins. In nearly all documented instances, the underlying pathophysiological mechanism was ascribed to mechanical compression of the pelvic venous system by a sizable or strategically positioned fibroid. The sites of compression predominantly included the common and external iliac veins and, in numerous reports, the inferior vena cava (IVC). The mass of the myomatous uterus exhibited considerable variability, with several specimens exceeding 5000 g, and larger fibroids were more commonly associated with thromboembolic events. Additional contributory factors included coexisting anatomical or hematological anomalies such as May–Thurner syndrome, intravenous leiomyomatosis, and myomatous erythrocytosis.

Numerous predisposing clinical factors have been repeatedly identified in the literature, most notably obesity, iron-deficiency anemia brought on by persistent uterine bleeding, and, to a lesser degree, the use of oral contraceptives. Dyslipidemia and antecedent thromboembolic events were also reported in a restricted subset of studies. None of the patients analyzed in the reviewed literature had any evidence of inherited thrombophilias. An intraluminal mass that spreads throughout the venous system and serves as a direct nidus for thrombus formation is less frequently caused by intravenous leiomyomatosis [23,35]. Additionally, severe uterine hemorrhage often resulted in iron-deficiency anemia in these individuals; hence, anemia may increase the risk of thrombosis by altering hemostasis and causing endothelial dysfunction [21]. Conversely, polycythemia may develop in a minority of cases, involving autonomous erythropoietin-producing tumors, a condition referred to as myomatous erythrocytosis, which results in a procoagulant state [33]. Inherited thrombophilias—such as Factor V Leiden—are established VTE risk factors in the general population, and they plausibly increase susceptibility in women with UFs [36]. It is interesting to note, nevertheless, that none of the examined case reports showed that the afflicted individuals had confirmed hereditary thrombophilia. Oral contraceptives raise the risk of thrombosis and have been linked to VTE in vulnerable people, according to a number of evidence from the literature [37]. Several cardiometabolic comorbidities, including obesity and dyslipidemia, were repeatedly noted as potential modifying risk factors. These conditions may act through endothelial dysfunction, chronic inflammation and altered hemostatic balance to increase susceptibility to VTE when pelvic venous stasis is present [38]. Although dyslipidemia and obesity are recognized risk factors for venous thromboembolism, they were observed in only one patient in our review. Obesity has been established as an independent risk factor for VTE, with several studies demonstrating a two- to threefold increased risk of both first and recurrent thromboembolic events in obese individuals, while a reduced risk has been reported among underweight patients [39,40]. Higher body mass index is strongly correlated with an increased risk of thrombosis, showing a clear dose-dependent relationship across categories of obesity [41]. Similarly, dyslipidemia has been associated with increased venous thrombosis risk, with multivariable analyses demonstrating significantly higher odds of VTE in individuals with abnormal lipid profiles [42]. The relatively low number of obese or dyslipidemic patients in our cohort may reflect the demographic characteristics of women with uterine fibroids who are often of reproductive age, whereas both dyslipidemia and obesity are more prevalent in older populations. Nevertheless, considering the global rise in obesity prevalence—particularly in developed countries—an increase in thromboembolic events among women with fibroids may be expected in the future [41,43].

The majority of documented DVT patients had unilateral leg discomfort and swelling. Venous compression most commonly happened at the level of the common or external iliac vein in the cases examined in this review, however the IVC was also affected in a few instances. The deep veins of the lower limbs, specifically the femoral and popliteal veins, were most frequently impacted by thrombosis and subsequent blockage; the iliac veins were involved in fewer instances. It is interesting to note that DVT mostly affected the left leg, and the results from the examined reports supported this laterality [37]. With respect to lateralization, left-sided lower-limb venous thrombosis was documented in 10 of 24 cases (41.7%), whereas right-sided thrombosis was observed in 2 patients (8.3%). Bilateral lower-limb involvement was reported in 1 case (4.2%). In the remaining 11 cases (45.8%), thrombosis involved central venous structures such as the inferior vena cava or the side of lower-limb involvement was not specified.

**Figure 2:**
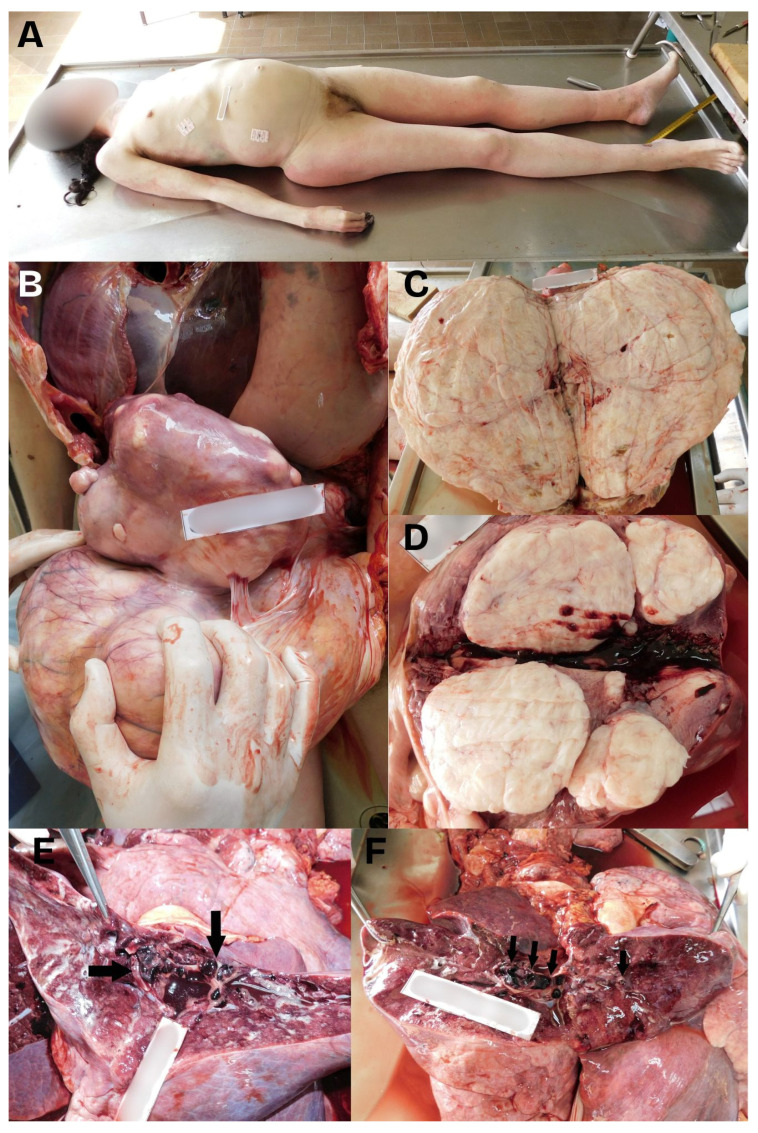
A composed image showing: (**A**) The cadaver of a 40-year-old woman showing a light protrusion of the abdomen above the level of the thorax. (**B**) The autopsy revealed significantly enlarged uterus, which weighed 9600 g. The uterus had a significantly thinned wall and was firm in consistency, with many myomas. The enlarged uterus compressed organs of the abdominal and thoracic cavities upwards. (**C**,**D**) The largest myoma was about 30 cm in diameter, with a number of smaller tumors with a diameter of 1 cm up to the size of a male fist. Some of the smaller myomas protruded into the uterus cavity. (**E**,**F**) The autopsy of the lungs revealed massive thromboembolism in both lungs, with both main pulmonary arteries and their lobar and smaller branches being comprising emboli (black arrows).

The main risk associated with deep vein thrombosis was embolization, which can be fatal if a dislodged thrombus passes through the right heart and blocks the pulmonary arteries (Figure 2). Pulmonary embolism developed in a portion of individuals, sometimes as the initial clinical sign. These occurrences usually happen quickly, creating an acute clinical picture that, if left untreated, can be lethal [44]. Among the 24 reported cases included in the review, isolated deep vein thrombosis was observed in 7 patients (29.2%), while isolated pulmonary embolism was documented in 4 patients (16.7%). Concomitant deep vein thrombosis and pulmonary embolism were present in 8 cases (33.3%). The remaining 5 cases (20.8%) involved other thrombotic manifestations and were not classified as classical deep vein thrombosis or pulmonary embolism. In half of the cases, patients had pulmonary embolism, confirmed by imaging methods.

All documented cases of thromboembolism had favorable outcomes since the symptoms were identified early and the right treatment was given right away. The first-line treatment for PE and DVT is still anticoagulant medication [26]. However, because of the myomatous uterus’ pressure and compression on the pelvic blood vessels, hysterectomy was eventually carried out in most patients. Since all symptoms were alleviated and the chance of additional thromboembolic consequences was reduced, this treatment strategy turned out to be the most successful [45]. Prior to surgery, IVC filters were placed (or balloon occlusion was performed) to prevent the migration of emboli from the lower extremity veins into the heart or pulmonary circulation [25]. In several cases, depending on individual risk assessment, thrombectomy or thrombolysis was also performed, prior to gynecologic surgery [26]. Of the 24 patients, 17 (70.83%) underwent hysterectomy, while three (12.5%) underwent myomectomy to preserve fertility and the possibility of future pregnancy. In the remaining four cases (16.67%), no gynecologic surgery was performed and only the thromboembolic event was managed—in two of these reports, hysterectomy was recommended but it was not specified whether the procedure was ultimately performed, whereas in the other two cases this information was not provided. Hysterectomy was deemed appropriate because most patients were perimenopausal and had no desire to become pregnant. Another instance included three younger women who wanted to become pregnant and underwent a uterus-preserving treatment like a myomectomy. These treatments were also successful, but unlike hysterectomy, there is still a chance of UF recurrence and, as a result, DVT [30]. All outcomes were favorable for the patients who were followed after their treatment, meaning no thromboembolic complications occurred, wherever such follow-up was reported. In patients who underwent myomectomy, no recurrence of fibroids was reported, and one patient subsequently had a successful pregnancy and delivered by cesarean section.

## 4. Discussion

### 4.1. Overview of Reported Cases and Pathophysiological Associations

We have compiled 24 reported cases of VTE associated with UFs from 18 studies to provide insight into possible causes, risk factors, and outcomes. Although uterine leiomyomas are common benign tumors with an underestimated potential to induce life-threatening thromboembolic effects, the examples collected here highlight the need for clinical monitoring when large or strategically placed fibroids are present. Menorrhagia and pelvic discomfort are common outcomes of UFs, which affect a sizable portion of women of reproductive age. Due to compression of surrounding structures (particularly pelvic veins), persistent blood loss that causes iron-deficiency anemia, and, in rare instances, intravascular extension, as in intravenous leiomyomatosis, large masses can result in substantial morbidity.

Abreu Martins and Sousa [19] reported a case of a 50-year-old woman with a single large fibroid (125 × 90 × 155 mm) compressing the iliac vessels, who developed DVT in the left popliteal vein, PE, and a cerebrovascular accident due to a patent foramen oval, and subsequently underwent total hysterectomy [19]. Alkhawam et al. described a 47-year-old patient with a large intramural fibroid (150 × 91 × 161 mm) causing bilateral iliac vein compression and resulting in bilateral calf-vein DVT and PE, followed by hysterectomy, after which the patient made a full recovery [20]. Almassinokiani et al. presented the case of a 37-year-old woman with three subserosal fibroids (largest 101 × 87 mm, weight 1500 g) complicated by chronic portal vein thrombosis, followed by hysterectomy, after which the patient made a full recovery [21]. Batista and Antunes reported a 47-year-old woman with compression of the left iliac vein leading to PE, who, after the embolism was adequately treated with anticoagulants, underwent hysterectomy, after which thromboembolic events did not recur [22]. Brown and colleagues described two cases: a 33-year-old woman with three intramural fibroids, bilateral external iliac vein compression, PE, and femoral/iliac DVT, who was treated solely with anticoagulants and thrombolysis; and a 37-year-old woman with 17 fibroids, which were removed by elective myomectomy, postoperatively developed pulmonary embolism, leaving her in critical condition (clinically dead for up to 15 min). She underwent thrombolysis and surgical thrombectomy, and due to significant uterine bleeding, uterine artery embolization was also performed [17]. Dong et al. reported a 43-year-old patient with a large submucosal fibroid weighing 5620 g, compressing the left iliac vein and IVC. Her chief symptom was a prolapse of tumor from the perineum, complicated by infection. The case was further complicated by multiple bilateral PE, DVT, acute cardiac and renal insufficiency, and shock. Management included open hysterectomy and perioperative anticoagulation. The patient tolerated the perioperative period well and made a full recovery [23]. Gil et al. presented a 35-year-old woman with multiple fibroids (195 × 186 × 120 mm) compressing the iliac veins and IVC, who experienced recurrent unprovoked thrombotic episodes and eventually developed PE. She was treated with anticoagulants and advised to undergo hysterectomy [24]. Kotsis et al. described a 46-year-old patient with a very large fibroid (270 × 180 × 155 mm) compressing the IVC and causing iliofemoral deep vein thrombosis, and prior to hysterectomy, balloon occlusion of the IVC was performed to prevent intraoperative PE [25]. Lacharite-Roberge et al. reported five cases of women aged 35–57 years, most of whom had multiple fibroids causing significant compression of the IVC or iliac veins; all had chronic thromboembolic disease and chronic thromboembolic pulmonary hypertension. All patients underwent pulmonary thromboendarterectomy, which resulted in immediate hemodynamic improvement, and after a period of follow-up, three of the five patients also underwent hysterectomy, after which none experienced further thromboembolic events [16]. Maruyama and Miyamoto presented a 71-year-old woman with a large fibroid (230 × 220 × 85 mm) compressing the IVC and left common iliac vein, who developed iliofemoral DVT, further promoted by the presence of May–Thurner syndrome. The patient was treated with anticoagulants and underwent hysterectomy, after which no thromboembolic complications were observed during the two-year follow-up period [26]. Nartey et al. reported a 40-year-old woman with two intramural fibroids who developed DVT and PE, was treated with anticoagulants, and subsequently underwent hysterectomy. The authors highlight the challenges of managing this condition in resource-limited settings [27]. Qammar et al. described a 38-year-old patient with multiple fibroids (ranging from 438 to 2231 g) who developed left-sided iliac, femoral, and popliteal DVT and PE, for which thrombectomy was performed, followed by anticoagulation therapy and subsequent hysterectomy, after which the patient made a complete recovery [28]. Qureshy et al. reported a 49-year-old woman with a massive lipoleiomyoma weighing 10,200 g that compressed the IVC and common iliac veins, leading to DVT for which thrombectomy was performed. Postoperatively, however, she developed “phlegmasia cerulea dolens”, necessitating fasciotomy as the initial intervention. In the subsequent course of treatment, she also underwent hysterectomy, preceded by bilateral uterine artery embolization to minimize intraoperative blood loss [29]. Sangeethaa and Abeysekara presented a 30-year-old woman with an anterior intramural fibroid causing ureteral compression and subsequent iliofemoral DVT. Given her desire to preserve fertility, myomectomy was chosen as the treatment approach in this case [30]. Speranza et al. described a 43-year-old woman who developed May–Thurner syndrome secondary to compression from a large uterine fibroid, which led to extensive DVT involving the IVC and iliofemoral veins, and subsequently PE. After thrombolysis and hysterectomy, the patient made a full recovery [31]. Tinawi et al. reported a 48-year-old patient with multiple fibroids compressing the IVC and femoral veins, leading to DVT, PE, and subsequent limb-threatening compartment syndrome that resulted in severe hyperkalemia due to rhabdomyolysis and the need for renal replacement therapy. She underwent thrombectomy, fasciotomy, hysterectomy, and small bowel resection due to necrosis and perforation [32]. Valente et al. described a 44-year-old woman with a large intramural fibroid (220 mm) who developed PE and venous sinus thrombosis due to myomatous erythrocytosis. Phlebotomy provided only a temporary solution, whereas following uterine artery embolization, her hematologic parameters stabilized. However, due to abdominal distension, the patient subsequently underwent hysterectomy [33]. Finally, Worrall and colleagues presented two cases: a 45-year-old woman with multiple fibroids compressing the left external iliac vein who developed iliofemoral DVT in the setting of prior DVT. She fully recovered following anticoagulant therapy and hysterectomy. The second case involved a 38-year-old woman with a large fibroid (240 × 100 × 170 mm) compressing the IVC and left common iliac vein, who developed extensive iliofemoral DVT. After adequate anticoagulant therapy, she underwent myomectomy, and later in life, she successfully delivered a child via cesarean section, demonstrating preservation of her fertility [34].

However, the majority of UFs remain asymptomatic or only cause local discomfort. In patients who are vulnerable, any of these factors can lead to venous stasis and thrombosis [35,46]. The ages of the women among the reported cases ranged from 30 to 71 years, with a mean age of 44.4 years. Across the reports, the predominant mechanism of VTE was mechanical compression of pelvic veins by a mass effect, producing venous stasis that predisposes to thrombus formation. This is further supported by the fact that thrombotic events are considerably more common in cases of large UFs, particularly those weighing more than 1000 g [47]. In the research we looked at, the weight of the UFs ranged from 1500 g to over 10,000 g. However, concurrent anatomic vascular abnormalities of the pelvis have been recorded in several cases, most notably May-Thurner syndrome, in which the left common iliac vein is compressed by the right common iliac artery. This means that the UFs themselves may not always need to apply compression directly. When fibroids are present, this arrangement may encourage the development of thrombus. Three cases where this condition was discovered in addition to UFs and thrombosis were included in this review [22,26,31].

Case reports that describe thrombosis after oral contraceptive therapy—which was prescribed to individuals with UFs, among other reasons—are not included in this analysis. This makes identifying the precise origin of thrombus formation extremely difficult [48,49].

Lacharité-Roberge et al. described seven cases of women with UFs who developed chronic thromboembolic pulmonary hypertension [16]. In addition to DVT and PE, a patent foramen oval in the case described by Abreu and Sousa resulted in cerebral artery blockage, which permitted paradoxical embolization—a very uncommon but clinically relevant occurrence [19]. Neurological symptoms were also reported in a case of venous sinus thrombosis, associated with polycythemia, secondary to myomatous erythrocytosis [33]. Almassinokiani et al. reported an extremely rare case of chronic portal vein thrombosis associated with UFs [21]. Compared to thrombosis of other veins, portal vein thrombosis in the presence of big myomas is thought to occur far less frequently. Two patients with UFs in this review had “phlegmasia cerulea dolens”, another serious but rare condition that necessitated an emergency fasciotomy [29,50].

### 4.2. Uterine Fibroids’ Treatment

UFs can be treated in several ways, from routine clinical surveillance to extremely invasive and drastic surgical operations. The size and location of the tumor, the patient’s age, the symptoms, and whether fertility preservation and the potential for pregnancy are significant post-therapy considerations all play a major role in the choice of treatment approach. An expectant approach is reasonable to apply for asymptomatic patients, as studies suggest that the size of the tumor decreases in menopausal women even without therapy. Additionally, a small percentage of cases have shown that fibroids may spontaneously regress within a few months in premenopausal women [51].

The management strategies were informed by the etiological factors and the severity of the thrombotic events. Anticoagulant therapy constituted the fundamental approach to initial management, whereas surgical interventions—most frequently hysterectomy—were ultimately executed in the majority of instances. In select high-risk patients, supplementary interventional procedures were conducted, encompassing IVC filter placement, catheter-directed thrombolysis, and endovascular or surgical thrombectomy. Myomectomy was specifically designated for younger women desiring to maintain their fertility and was associated with favorable outcomes, despite the persisting risk of recurrence. Overall, the prognosis was deemed favorable in all documented cases that had follow-up data available.

Medical treatment is also an option when fertility preservation is prioritized and when delaying surgical intervention is necessary [52]. In some cases, it can be used prior to surgery to reduce the size of the fibroid. The positive aspects of this approach include symptom reduction or even complete symptom resolution in many cases, especially a reduction in blood loss. However, a major downside is that almost none of these medications are suitable for long-term use [51,53]. The targets of pharmacological therapy are primarily hormones—estrogen, progesterone, and their receptors. [53]. As with any medication, there are potential side effects, and in the case of pharmacological treatment for UFs, especially combined oral contraceptives and Selective Estrogen Receptor Modulators in particular, are known to increase the risk of venous thrombosis [54,55].

Minimally invasive techniques are also used when preserving fertility is a top priority and when traditional surgical intervention is not practical. Radiofrequency ablation (RFA), magnetic resonance-guided focused ultrasound (MRgFUS), and uterine artery embolization (UAE) are examples of minimally invasive techniques. The process of uterine artery embolization results in controlled ischemia and eventual fibroid tissue necrosis, yet supplementary blood supply keeps the remainder of the uterus intact. As previously stated, this technique is used to preserve fertility; however, there have been reports of problems with reproductive function as well as pregnancy complications such fetoplacental insufficiency, which can result in fetal development limitation and even preterm birth. Therefore, UAE is not recommended for patients planning pregnancy after treatment [53].

Finally, myomectomy and, as a last resort, hysterectomy are invasive surgeries. A hysterectomy resolves symptoms permanently and removes the possibility of recurrence in the future. It is typically done as a last resort, especially for women whose fertility preservation is not a major issue. This surgery can be carried out in a number of ways, the most popular being vaginal and laparoscopic. Although there are size restrictions, laparoscopic techniques are frequently advised since the size of the uterus and fibroids greatly affect the viability of a vaginal approach; in some situations, laparotomy is the only option. In some cases, morcellation—the mechanical fragmentation of uterine tissue—can help conduct a laparoscopic hysterectomy even for a very big uterus, to facilitate easier removal. A laparotomy would eliminate the possibility of iatrogenic dissemination of potentially cancerous cells in the abdomen. Open hysterectomy, on the other hand, is much more invasive and has a higher risk of complications, including wound infection, thrombosis, and considerable blood loss. Myomectomy is a surgical procedure that permits future conception since the fibroids are removed and the uterus is rebuilt. Complications are rare, although thromboembolism may occur in this case [53].

### 4.3. Mechanism of Thromboembolism Development During Uterine Fibroid Surgery

In women with UFs, VTE is extremely uncommon, especially when there are no other thrombosis predispositions. It is well known that Virchow’s triad—hypercoagulability, venous stasis, and endothelial injury—offers a concise description of the processes by which thrombi arise. Venous stasis, which is caused by compression of the pelvic and lower extremities blood vessels, particularly by big fibroids, has been proposed as the most likely contributing factor in the case of UFs [28].

Some researchers further stress that, in contrast to the consistently expanded uterus encountered during pregnancy, the likelihood of VTE is considerably higher when the uterus is irregularly inflated. However, polycythemia and reactive thrombocytosis, which both contribute to a hypercoagulable state, can result from the frequent bleeding that characterizes UF. Menorrhagia is also a common reason to use tranexamic acid or oral contraceptives, both of which are procoagulant medications [56].

Although relatively few cases have been reported, the risk of VTE is still a worry when it comes to surgical treatment of UF. Research has shown that pelvic surgeries are more often linked to thromboembolic events than procedures in other locations, and that DVT occurs in almost 30% of cases in the absence of sufficient preoperative anticoagulation therapy, compared to only 0.2% in those who receive the proper prophylactic treatment [18]. After a hysterectomy and the removal of UFs that clog the veins, women with DVT who do not exhibit clinical symptoms may experience an embolism if previously created thrombi move to the pulmonary circulation [56]. VTE may also occur if heparin administration is interrupted during surgery [57].

Because laparoscopic gynecological operations, such as myomectomy, are less invasive, require less recovery time, and allow for a quicker return to normal activities, they are known to lower the risk of VTE when compared to open surgery. Even if the operation is minimally invasive, an extended duration greatly raises the risk. This risk is further increased by additional factors like obesity or the existence of cancer [58].

Because there are less surgical trauma and less need for retractors, laparoscopies are less risky than laparotomies. However, they frequently take longer and involve higher intra-abdominal pressure because of pneumoperitoneum, which can compress blood vessels and cause venous stasis. The Trendelenburg position, which is frequently used in most gynecological laparoscopic procedures, can also result in stasis. Despite the fact that laparoscopic surgery is linked to shorter hospital stays and postoperative recovery times, it has been noted that these patients are not as easily moved at home [59].

The review’s distinctiveness, which tackles a subject that is crucial for its influence on patients’ health but has received little attention in the literature, is the study’s strongest point. The primary drawback, though, is the paucity of studies we could locate on the subject.

## 5. Conclusions

VTE linked to UFs can result in significant morbidity, despite being rare. The primary mechanism is mechanical venous compression, which is frequently compounded by other prothrombotic variables. When VTE remains unexplained, clinicians should explore early surgical consultation to address compressive etiologies, maintain a low threshold for venous imaging in women with significant pelvic masses and unilateral limb complaints, and check for concomitant thrombophilia. Standardized reporting of fibroid-associated VTE and prospective registries would make it easier to determine the true incidence and identify risk factors that can be changed.

## Figures and Tables

**Table 1:** Overview of previously reported cases of uterine fibroid-related thromboembolism.

Author	Year	Pt	Age, Years	Fibroid *	Wt (g)	Compression Site	Presentation	DVT	Thrombophilia	Risk Factors	Treatment	Outcome	Investigated Population
Abreu Martins and Sousa [19]	2022	1	50	one, 125 × 90 × 155 mm	NR	iliac vessels	DVT, PE, CVA	L popliteal	No	obesity, dyslipidemia	EVT, hyst, AC	favorable	Portugal, NR
Alkhawam et al. [20]	2024	1	47	one, 150 × 91 × 161 mm, intramural	NR	BL iliac veins	DVT, PE	R posterior tibial, peroneal, L tibioperoneal trunk	NR	obesity, IDA	lysis, hyst, AC	favorable	USA, NR
Almassinokiani et al. [21]	2022	1	37	three, subserosal: 101 × 87 mm (posterior) 3 × 21 mm (anterior) 26 × 22 mm (anterior)	1500	NR	CPVT	NR	No	IDA	hyst, AC	favorable	Iran, NR
Batista and Antunes [22]	2022	1	47	NR	NR	L iliac vein	PE	L iliac vein	No	obesity, OCD, MTS	hyst, AC	NR	Portugal, NR
Brown et al. [17]	2024	1	33	three, 93 × 83 × 77 mm (posterior) 42 × 40 × 30 mm (posterior) 12 × 8 × 10 mm (anterior), intramural	NR	BL external iliac veins	PE	R femoral and external iliac	No	NR	lysis, AC	NR	USA, NR
		2	37	multiple (17)	NR	NR	PE	NR	No	IDA	lysis, surg thromb, IVCF, myomectomy, UAE	NR	
Dong et al. [23]	2024	1	43	one, 200 × 150 × 100 mm, submucosal	5620	L iliac vein, IVC	DVT, PE	L femoral, popliteal, posterior tibial, peroneal	NR	IDA, history of DVT	IVCF, AC, hyst	favorable	China, NR
Gil et al. [24]	2025	1	35	multiple, 195 × 186 × 120 mm	NR	Iliac veins, IVC	DVT, PE	NR	No	IDA, obesity, history of unprovoked PE	AC	NR	USA, NR
Kotsis et al. [25]	2022	1	46	one, 270 × 180 × 155 mm	NR	IVC, gallbladder	DVT	L iliac-femoral	NR	NR	IVC baloo occlusion, hyst	NR	Greece, NR
Lacharite-Roberge et al. [16]	2019	1	48	one, 204 × 173 × 202 mm	NR	IVC, BL common iliac veins	DVT	IVC	NR	PMH significant for DVT and PE	PTE, hyst	favorable	USA, Caucasian
		2	53	multiple, largest: 80 × 65 × 54 mm	NR	external iliac vein	NR	NR	NR	PMH significant for DVT and PE	PTE	favorable	USA, African American
		3	35	multiple, largest: 56 mm	NR	BL external iliac veins	NR	NR	NR	PMH significant for DVT and PE	PTE, hyst	favorable	USA, NR
		4	52	three, largest: 58 × 32 mm	NR	IVC, BL common iliac veins	NR	NR	NR	PMH significant for DVT and PE	PTE, hyst	favorable	USA, NR
		5	57	multiple, largest: 15 × 10 mm	NR	NR	NR	NR	NR	PMH significant for DVT and PE	PTE	favorable	USA, African American
Maruyama and Miyamoto [26]	2020	1	71	one, 230 × 220 × 85 mm	2060	IVC, L common iliac vein	DVT	iliofemoral	No	MTS	IVCF, AC, hyst	NR	Japan, NR
Nartey et al. [27]	2020	1	40	two, 64.8 × 54.5 × 71.2 mm (anterior) 29.5 × 28.4 × 22.3 mm (posterior), intramural	NR	NR	DVT, PE	R femoral, popliteal	NR	OCD	AC, hyst	NR	Ghana, NR
Qammar et al. [28]	2024	1	38	multiple, 210 × 170 × 115 mm	smallest 438, biggest 2231	NR	DVT, PE	L iliac, femoral, popliteal	No	obesity, IDA	EVT, AC, hyst	favorable	Nepal/Pakistan, NR
Qureshy et al. [29]	2025	1	49	NR	10,200	IVC, BL common iliac veins	DVT, PCD	L femoral	NR	No	EVT, AC, UAE, hyst	favorable	USA, NR
Sangeethaa and Abeysekara [30]	2023	1	30	multiple, anterior, intramural	NR	R ureter	DVT	L femoral, popliteal	NR	NR	IVCF, AC, myomectomy	favorable	Sri Lanka, NR
Speranza et al. [31]	2021	1	43	multiple	NR	NR	DVT, PE	IVC, iliofemoral	NR	OCD, MTS	AC IVCF, lysis, hyst	favorable	USA, Caucasian
Tinawi et al. [32]	2020	1	48	multiple, 123 × 144 mm	NR	IVC, BL femoral vein R external iliac vessels	DVT, PE, PCD	L iliofemoral, popliteal, posterior tibial	No	obesity	AC, IVCF, hyst	NR	USA, NR
Valente et al. [33]	2024	1	44	one, 220 mm, intramural	2022	N/A	PE, VST	N/A	No	OCD, ME	phlebotomy, AC, UAE, hyst	favorable	USA, Caucasian
Worrall et al. [34]	2024	1	45	multiple, largest: 38 × 47 × 44 mm	NR	L external iliac vein	DVT	L iliofemoral	NR	IDA, PMH significant fot DVT	AC, hyst	favorable	Ireland, Caucasian
		2	38	one, 240 × 100 × 170 mm	NR	IVC, L common iliac vein	DVT	L common and external iliac, femoral, popliteal	NR	OCD	AC, lysis, IVCF, myomectomy	favorable	Ireland, Caucasian

* Characteristic (number, size, location). Legend. Wt—weight; DVT—deep venous thrombosis; PE—pulmonary embolism; PCD—phlegmasia cerulea dolens; CVA—cerebrovascular accident; CPVT—chronic portal vein thrombosis; surg thromb—surgical venous thrombectomy; lysis—catheter-directed thrombolysis; EVT—endovascular thrombectomy; hyst—hysterectomy; L—left; R—right; BL—bilateral; NR—not reported; Pt—patient; IVC—inferior vena cava; IVCF—inferior vena cava filter; IDA—iron deficiency anemia; AC—anticoagulation; N/A—not applicable; UAE—uterine artery embolization; OCD—oral contraceptive drugs; MTS—May-Turner syndrome; ME—myomatous erythrocytosis; PTE—pulmonary thromboendarterectomy.

## Data Availability

The data generated by the study are available in scientific literature.

## Abbreviations

The following abbreviations are used in this manuscript:

UF	Uterine Fibroids
BMI	Body Mass Index
HRQL	Health-Related Quality of Life
UAE	Uterine Artery Embolization
MRgFUS	Magnetic Resonance-Guided Focused Ultrasound
VTE	Venous Thromboembolism
DVT	Deep Vein Thrombosis
PE	Pulmonary Embolism
IVC	Inferior Vena Cava
